# AI-Enhanced IoT System for Assessing Bridge Deflection in Drive-By Conditions

**DOI:** 10.3390/s25010158

**Published:** 2024-12-30

**Authors:** Leonardo Iacussi, Paolo Chiariotti, Alfredo Cigada

**Affiliations:** Department of Mechanical Engineering, Politecnico di Milano, Via Privata Giuseppe la Masa 1, 20156 Milano, Italy; leonardo.iacussi@polimi.it (L.I.); alfredo.cigada@polimi.it (A.C.)

**Keywords:** intelligent sensors, indirect SHM, MEMS sensors, edge AI, IoT infrastructure

## Abstract

The increasing traffic on roads poses a significant challenge to the structural integrity of bridges and viaducts. Indirect structural monitoring offers a cost-effective and efficient solution for monitoring multiple infrastructures. The presented work aims to explore new sensing strategies based on digital MEMS sensors integrated into an intelligent IoT infrastructure to predict the bridge deflection behaviour for indirect Bridge Structural Health Monitoring purposes. An experimental setup comprising a bridge model and vehicle equipped with a smart sensing node has been used to generate the dataset. Various models for bridge deflection estimation are deployed on the sensorized vehicle, exploiting edge AI capabilities of smart sensors. This study shows the potential of leveraging data-driven technologies to enhance the performance of low-cost sensors. Additionally, it demonstrates the viability of assessing static deflection shapes of bridges through indirect measurements on board vehicles, underlining the potential of this approach to make SHM more cost-effective and scalable.

## 1. Introduction

Over the last few decades, there has been a noticeable increase in road traffic worldwide [1,2], accompanied by a significant escalation in the size and mass of vehicles, particularly in the context of trucks and heavy-duty vehicles. This trend has resulted in increased demand for safety assessments of transportation infrastructures, particularly bridges and viaducts. In fact, the increased load acting on aging bridge structures has the potential to exceed the originally designed capacities of the bridge, exposing the infrastructure to increased deformations and the possibility of unforeseen damages [3,4]. In the United States, the average age of infrastructure in poor condition is over 50 years, and an increasing trend of damaged infrastructure has been reported, noting that by 2023 there will be a total of 167 million vehicles crossing 42,400 bridges in poor condition every day [5]. In Europe, over 30% of current highway bridges are considered structurally deficient, with an even higher percentage when considering all short to medium span bridges [6,7]. Statistics from 2019 report that 47% of the 8165 TEN-T bridges in Germany are older than 40 years, and the same situation can be observed in other European countries such as Denmark and Portugal. In France, 26% of the bridges were built between 1951 and 1975 and are reaching the end of their design life [8,9].

Consequently, in recent years, there has been increasing concern regarding the safety of bridges and infrastructures in general. Both United States [10,11] and Europe [12] are actively contributing to a growing understanding regarding safety, maintenance, and the implementation of SHM systems in transport infrastructures. The most common approach to verify infrastructure integrity and ensure the required safety levels involves periodic visual inspections. However, this method is associated with challenges such as timing constraints and the subjectivity of inspection with respect to the assessment of the health condition of the target structure. Additionally, as infrastructure degradation progresses, more frequent visual inspections become necessary, leading to an escalation in maintenance costs [13]. At present, only a limited number of new bridges incorporate an integrated SHM system that provides continuous feedback on structural integrity. The main reasons for such a small percentage of monitored bridges are the high installation and maintenance costs [11]. A step change is therefore necessary to handle this issue properly. The exploitation of new technology in sensors, vehicles, connectivity, and big data becomes fundamental to make structural monitoring more accessible and scalable. This can be possible by combining new monitoring techniques, such as indirect Bridge Structural Health Monitoring (iBSHM), Crowdsensing techniques and by using new sensor technologies to create more scalable intelligent sensor networks. Conventional structural monitoring solutions involve the installation of various sensors directly on the infrastructure. The sensors are used to study the dynamic or static response of the infrastructure, depending on the sensors and damage identification algorithms to be used [14,15]. As previously mentioned, monitoring infrastructure through structural monitoring solutions can be challenging to scale up. This particularly true when planning SHM interventions on old and structurally deficient structures [16]. Therefore, indirect structural health monitoring aims to use the passage of vehicles equipped with appropriate sensors on bridges to extract parameters related to the health status of the bridge. Each vehicle can potentially contribute to monitoring multiple infrastructures on a regular basis through indirect structural monitoring. The advantages in terms of ease of deployment and cost-effectiveness are clear on a single bridge. However, vehicles potentially work as mobile scanners during the measurement process, paving the way to more accurate damage location on the structure. This feature proves advantageous in situations where deploying a dense network of sensors directly onto the infrastructure is impractical or economically unfeasible. This technology will be increasingly exploitable with the spread of new intelligent and connected vehicles, leveraging on vehicle to infrastructure (V2I) communication as well as on algorithms and methodologies for managing big amounts of collected data. In the existing body of literature, the majority of methodologies used for indirect structural monitoring primarily involve the analysis of acceleration signals obtained onboard a vehicle. These signals are typically analyzed in either the time or frequency domain, aiming to extract essential structural information such as natural frequencies and associated mode shapes, induced by vehicular traffic. Other studies use the signals coming from vehicles to extract the instantaneous curvature (IC) or track irregularities of the bridge in order to find and localize structural damages [17,18,19]. Notably, some research endeavors have concentrated on the analysis of a single vehicle passage, while others have delved into amalgamating information from multiple vehicles. The latter approach harnesses data redundancy to enhance precision in extracting structural information, which is the main purpose of crowdsensing techniques for SHM [20]. Singh, as discussed in [21], thoroughly examines the necessary avenues for advancing iBSHM. One of the foremost challenges involves the critical need to develop an appropriate vehicle model. This requirement stems from the fact that acceleration measurements taken aboard the vehicle are subject to filtration by suspensions and can be influenced by varying asphalt roughness and vehicle speed. Furthermore, ongoing inquiries persist regarding the communication and integration of emerging wireless sensor networks within an Internet of Things (IoT) infrastructure. This entails the intricate management of the substantial volume of data collected by the comprehensive sensor network, addressing both architectural and algorithmic considerations.

### 1.1. Smart Sensors

In the past few years, the widespread adoption of wireless sensor networks and the omnipresence of the IoT have generated a need for sensor technologies that go beyond traditional solutions. As these networks expand to cover diverse applications, the importance of intelligent and adaptable sensors continues to grow also in the field of SHM, enhancing the possibility to have continuous monitoring at lower cost and with an higher responsiveness [22]. Modern SHM systems embed several sensing nodes deployed on the target structure with a centralized gateway which is able to collect raw data (e.g., vibrations, temperature, strain) from the field devices and to share them on a cloud platform for storage and analysis purposes. Considering a scenario where a dense sensor network is deployed on a bridge, or vehicles in case of iBSHM, the communication and storage capabilities at the cloud level will be of major concern in terms of network traffic, cost, energy consumption and scalability [23]. Consequently, by embedding microcontrollers and Micro Electro-Mechanical Systems (MEMS)-based digital sensors in sensing nodes, it is possible to completely rethink the IoT architecture by dividing the computational load of processing algorithms on different levels: on the sensor itself [24], on the sensing node (thus turning it into a smart sensing node), at the gateway level and eventually on the cloud [25]. The use of edge-processing and edge Artificial Intelligence (AI) is the basis of these new architectures, making it possible to greatly reduce data traffic between sensors and the cloud by processing data directly on the sensor and sending only higher-level information instead of raw sensor data. Computational power on microcontrollers is limited, but with today new technologies it is possible to run artificial intelligence algorithms on the smart sensing node itself, using appropriate model quantization strategies or specific training approaches (e.g., federated learning [26]). If the computational power required for the algorithm turns out to be insufficient, gateways, now also equipped with GPUs to speed up complex computations, can be used to agglomerate information from different sensing nodes and make predictions through models computationally more expensive. The cloud would then turn to be a platform for controlling the entire monitoring system and for visualizing the health of the infrastructure in real time.

However, there are several metrological aspects related to the use of these sensing nodes that often make them more challenging to use. The electrical noise of the sensors is often high, given their reduced sensitivity. It is therefore more difficult to identify those small variations in the signals that characterize possible damages [27]. By leveraging the potential of crowdsensing and the large amount of redundant collected data, it is possible to statistically reduce the uncertainty on the measurement due to low sensitivity [28].

An additional concern associated with the deployment of an extensive wireless sensor network pertains to the synchronization of diverse sensing nodes, which may lack sufficient accuracy for certain application scenarios. Various synchronization strategies, contingent upon different communication systems, offer differing degrees of accuracy in achieving synchronization (e.g., Network Time Protocol [29]). In the context of iBSHM, synchronization among all devices is not always a strict requirement if each vehicle passage may be considered independently. Nevertheless, this assertion does not consistently hold true when contemplating models that leverage crowdsensing techniques using data from concurrently crossing vehicles on the structure, wherein the temporal coherence of the dataset proves pivotal for the application of some algorithms [30]. A thorough understanding of the capabilities and limitations of these sensors is essential for creating a coherent and robust dataset that enables effective modeling of the phenomenon.

### 1.2. Presented Research Work

**Figure 1:**
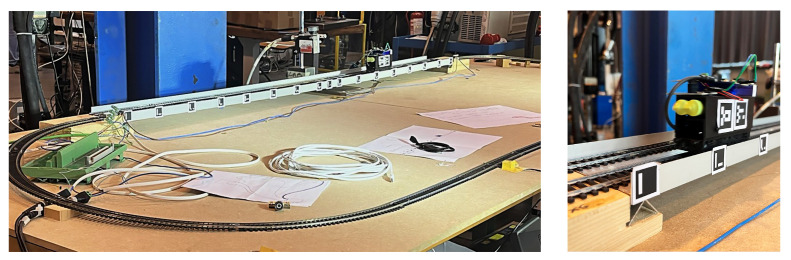
(**left**) Experimental setup. (**right**) Sensorized vehicle.

The objective of this work is to demonstrate the enhanced possibilities of SHM Systems grounding on AI-supported IoT architectures for indirect bridge monitoring. This approach can enhance the scalability of the system across multiple infrastructures and significantly reduce the SHM cost as presented before. In particular, this paper aims to demonstrate the feasibility of assessing the static deflection shape of a bridge through indirect measurements using vehicles passing over within a smart infrastructure ecosystem. To the authors’ knowledge, this approach has never been proposed so far, as more common techniques involve the use of clinometers installed on the bridge [31]. The focus is on the data acquisition and processing architecture, with partial utilization of edge computing for the calculation of the static bridge deflection. Within the framework of this research, a laboratory experimental setup is implemented (see Figure 1), exploiting an intelligent and connected vehicle model without suspension. Despite being a testing condition similar to the one involving train on railway bridges, the wheels and train underbody stiffness is high enough to consider the vibration path less affected by the dynamics of the vehicle than in cars and trucks. This intentional decision is made to divert attention from specific vehicle dynamic behaviour, with the primary objective of clarifying the fundamental principles of the IoT architecture and the application of intelligent sensors in the realm of indirect structural monitoring.

The paper is organized as follows. The second section describes the experimental setup used for the investigation. This includes the calibration of smart sensing nodes used to evaluate the vehicle angle, as well as the formulation of a mathematical model that simplifies the representation of bridge mechanics and vehicle-bridge interaction. The sections that follow compare the deflection of the bridge, as estimated by the moving sensing vehicle, with a reference sensor system and a mathematical model. This comparison aims to understand the error in estimating the bridge stiffness.

## 2. Materials and Method

This section provides a comprehensive description of the experimental setup used in this research, as well as the processing strategies adopted to calculate the deflection of the bridge from an IoT node installed on a vehicle passing over the bridge. More specifically, it discusses the main characteristics of the bridge model, the smart sensing nodes deployed, the IoT infrastructure that facilitates the sensor interaction and the reference data acquisition system. The theoretical mechanical model used to characterize the behavior of the vehicle-structure interaction is then presented. In the end, the data processing and algorithmic methodology used to extract the static deflection of the structure is discussed. Results will be presented in the last section of this work.

### 2.1. Experimental Setup

**Figure 2:**
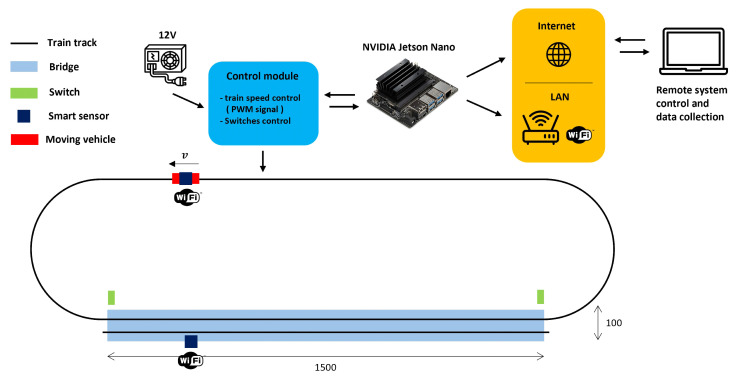
IoT Experimental setup schema.

The experimental set-up consists of a small-scale replica of an IoT architecture (see Figure 2) targeted to iBSHM. The architecture also takes into account the potential communication between vehicles and infrastructures, which started being available in certain real-world application cases. More specifically, the setup comprises an aluminum bridge model spanning 1500 mm, hosting a rail track wherein the vehicle speed can be controlled by adjusting the rail voltage value between 0–12 V. Data collection occurred concurrently using three distinct measurement systems. The first measurement systems grounds on two smart sensing nodes, deployed on the vehicle and the bridge, respectively. These nodes communicate with the infrastructure (the small scale bridge), collecting data from the internal Inertial Module Unit (IMU) and sharing them across the network via wireless communication. The second system exploits a laser triangulation displacement sensor to accurately capture mid-span displacement as the train traverses the bridge. The third system uses an industrial camera to monitor both the vehicle and bridge movements. This vision-based system leverages ArUco Markers placed on the bridge and the moving vehicle to measure the displacement of the bridge as well as track the location of the vehicle during the pass-over. Acquisition of data from the three systems occurs exclusively when the vehicle is on the bridge. This is made possible by switches located at each side of the bridge that trigger the acquisition of all signals.

#### Bridge Mechanical Properties

**Figure 3:**
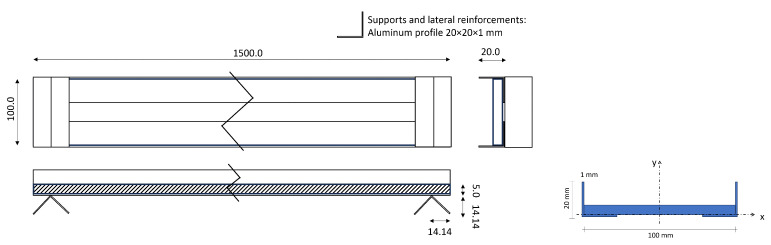
(**left**) Bridge geometrical dimensions. (**right**) Bridge deck geometrical dimensions.

The aluminum bridge model consists of a 1500 mm long, 100 mm wide, and 5 mm thick aluminum metal sheet. Additionally, it incorporates two longitudinal aluminum reinforcements formed by L-shaped aluminum trusses (cross-section dimensions: 20×20×1 mm), securely bonded to the aluminum metal sheet by two-component adhesive for aluminum (see Figure 3). The total weight of the bridge is 2.2970 kg, resulting in an average density of the material of ρ=2575 kg/m^3^. The Young Modulus considered in the calculation is *E* = 70,000 MPa. The vehicle used is a h0-scale train specifically modified to host a smart sensing node. The train total weight is 0.2732 kg: this results in a vehicle-bridge mass ratio of around 12%. Despite being quite high, this ratio represents a reasonable value for heavy vehicles. In an effort to emulate an ideal scenario, the bridge demonstrator is supported by two L-shaped profiles, mimicking pinned-pinned boundary conditions and resulting in a bridge free span of 1472 mm. While the pinned-pinned boundary condition might be not completely ideal, giving the presence of rail tracks crossing the bridge and being constrained to the bridge abutment, we are assuming that the pinned-pinned beam model effectively captures the bridge static and dynamic behavior. The detailed values regarding the deck section have been calculated with respect to the *x* and *y* axis, see Figure 3. The center of gravity is $y_{G}=2.75$ mm and $x_{G}=0$ mm, with respect to the geometrical symmetrical axis, and the bridge deck section inertia with respect to the *x* axis corresponds to $I_{xG}=4373.29$ mm^4^.

### 2.2. Smart Sensing Nodes

The IoT infrastructure is designed to emulate a real-world sensing strategy, encompassing different layers that consist of intelligent sensors nodes equipped with edge computation capabilities. The field device layer involves several smart sensors distributed along the bridge or installed on vehicles. These sensors feed data into a primary data aggregator system, made of a high-performance gateway, capable of executing complex algorithms. The smart sensing nodes deployed are derived from the STMicroelectronics STEVAL-STWINKT1B evaluation board [32]. This evaluation board integrates an Ultra-low-power Arm Cortex M4 32-bit microcontroller unit (MCU), specifically the STM32L4R9 [33], incorporating also a floting point unit (FPU) which enable fast digital signal processing calculations using hardware-dedicated software libraries (e.g., CMSIS-DSP [34]). Moreover, the board integrates many different MEMS-based sensors including inertial sensors, environmental sensors and microphones. The focus in this research is on the ISM330DHCX IMU [35], communicating via SPI with the microcotroller, in order to extract the sensor rotation angles leveraging the potential of the MEMS accelerometer to sense the gravity acceleration. The challenges associated with using a digital sensor, as opposed to an analog ones, show up in increased adaptability that takes full advantage of the sensor inherent computational capabilities, limited embedded memory and manageable output data rate (ODR) and full scale (FS). This sensor demonstrates the potential to execute tiny decision trees for event detection, further enhanced by its programmable low-pass and high-pass digital filtering capabilities. This has great potential to reduce the computational load on the associated microcontroller, which can then perform other operations, such as computation and transmission, or be put into sleep mode for low-power applications. Given the acceleration levels involved in this experiment, the FS has been set to ±2 g, which is the lowest FS available for the 16-bit digital sensor used. The ODR was set to 104 samples/s. The internal composite filter, place just after the anti aliasing filter, has been set as a low pass digital filter with a cutoff frequency of ODR/45 resulting in 2 Hz of pass-band bandwidth. The sensor has been used in FIFO+Interrupt mode leveraging the internal memory to store a buffer of 100 samples for the 3 axes as 16-bit acceleration filtered data before transmitting them to the microcontroller. Between receiving one buffer and the next, approximately every second, the microcontroller calculates the pitch and roll angles. It then transmits these angles via WiFi (IEEE 802.11 protocol) to the gateway, which responds with the current time stamp needed for synchronization, using the NTP protocol algorithm for clock synchronization between the gateway and each smart sensing node. The connectivity module is an external ESP8266 (ESP-01) WiFi module, connected using UART protocol to the available STDMOD+ pinout interface of the board. In this case, any real time operating system (RTOS) has been used on the microcontroller, but it could be included in the future to have a better control among the different tasks of the smart sensing node. The gateway is a NVIDIA Jetson Nano board [36] which incorporates a GPU suitable for speeding up Machine Learning or Artificial Intelligence approaches. It features two Network Interface Cards (NIC): one connected to a modem, establishing a Local Area Network (LAN) for handling the WiFi communication with the smart sensing nodes, and the other linked to internet for transmitting the collected data to a remote host (see Figure 2). The gateway runs an efficient multi-task Python code in which each task manages each connected smart sensing node by using a TCP-IP server-client multi threaded software architecture.

### 2.3. Bridge Mid-Span Displacement Sensor

A high performant laser triangulation displacement sensor was placed at the bridge mid-span, in order to have an accurate measurement of the maximum bridge displacement during the train passage serving as reference measurement. The output voltage of the sensor ranges from 1 V to 5 V corresponding to a full scale of 50 mm. It results in a sensitivity of 12.5 mm/V. The sensor was acquired using a 24-bit acquisition system over a range of 0–10 V giving a lest count error in the order of nanometer, 7.45 × 10^−6^ mm/LSB. The accuracy of this tool is more than sufficient for the case study under investigation.

### 2.4. Vision System

**Figure 4:**
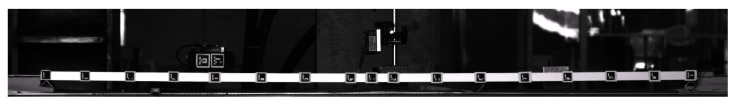
Camera record screen.

**Figure 5:**
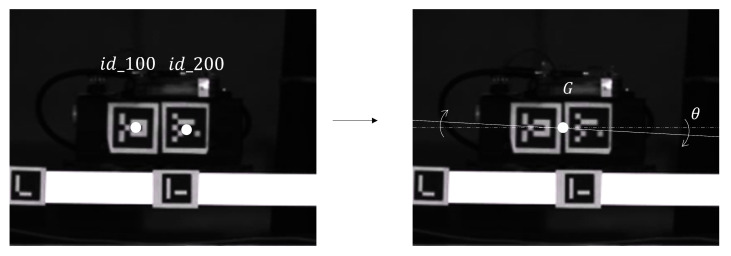
ArUco markers installed on the vehicle for displacement and angle calculations.

The vision-based system aims to provide detailed data on the vertical and angular tilting of the bridge with respect to the location of the train over the bridge. In addition, it is also intended to provide a more accurate estimate of the speed of the train along the bridge. This setup aims to assess changes in the train speed, even when the ideal voltage supply on its motor remains constant. To achieve this monitoring, 17 ArUco markers have been glued to the bridge structure (see Figure 4) while two further ArUco markers have been placed on the train in correspondence with its wheels (see Figure 5). The image sequences have been acquired using an industrial GiGE camera, specifically an Allied Vision Prosilica GX3300, equipped with a 50 mm f/2 lens (Zeiss Makro-planar T* ZF lens). This camera operates with a single 14-bit channel for image acquisition, offering a maximum resolution of 3296×2472 pixels. Given the slender nature of the structure, obtaining a significant number of vertical bits representing its movement poses challenges, even with a high-resolution camera. Consequently, the images are pre-processed by cropping them to a format of 3296×400 pixels before acquisition, allowing a maximum acquisition speed of 8 frames per second. The camera was calibrated using a checkerboard calibration target (squares on the side 20 mm) to compensate for image distortions and ensure consistent sensitivity across the entire frame. Before ArUco marker detection, each frame underwent the following preprocessing steps: (a) correction of image distortions (e.g., perspective distortions), (b) a clockwise rotation correction of 0.24 degrees, and upsampling with a 3× factor using bicubic interpolation. A sensitivity of 0.1748 mm/px was achieved. This value turned out to be adequate for calculating the displacements of the bridges at the target locations (ArUco marker locations). Subsequently, the markers were detected and their center of gravity was computed, facilitating the calculation of all displacements. In the model, the vehicle is treated as a rigid body, and its coordinates are positioned in the center of the vehicle. This involves consolidating the two centers of the ArUco markers into a single coordinate representing their average position. Subsequently, the relative angle between these markers is calculated and compared with the angle detected by the smart sensor. The Dewesoft SIRIUS (×8 IEPE channels) acquisition system and its management software DewesoftX were used to capture data from both the camera and the displacement laser sensor. This setup facilitates the synchronized acquisition of 8 analog channels and the GigE Ethernet camera. Triggering of the acquisition process at the initiation and conclusion points of the bridge has been achieved using switches. This configuration allows for the selective storage of pertinent data, effectively avoiding the accumulation of excessive and unnecessary data. The same two switches signals have been acquired also by the NVIDIA Gateway in order to correctly tag (i.e., train on-the-bridge vs. train off-the-bridge) the acceleration and the buffer data coming from the smart sensors. The sampling frequency used for the acquisition of the analog signals is 50 Hz, while for the GigE Camera, the maximum sampling frequency available for that resolution is 8 Hz, which provides sufficient time resolution for modeling this problem as quasi-static.

### 2.5. Smart Sensing Node Angle Calibration

**Figure 6:**
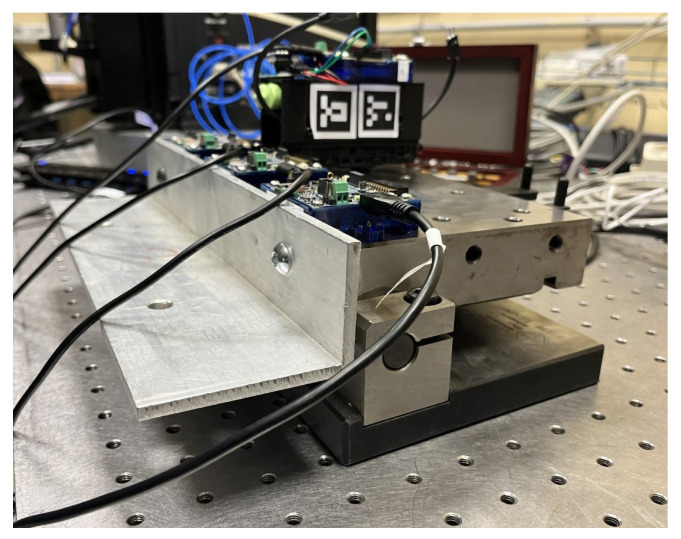
Smart sensing node angle calibration using a reference sine bar.

The determination of inclination angles is conventionally achieved by computing values through the utilization of MEMS-based 6-axis IMU sensors. These sensors have the capability to detect both gravity acceleration and angular velocity. Various algorithms have been developed to leverage the information derived from both acceleration and angular velocity in order to optimize the estimation of pitch and roll angles [37,38]. However, in the context of this paper, the decision was made to exclusively rely on the acceleration data obtained from the IMU. This choice stems from the fact that low-cost MEMS sensors often exhibit significant noise. Given the small angular velocities involved in this application if compared to changes in static acceleration values, introducing gyroscopic data would only amplify the noise in the calculation of roll and pitch angles. Consequently, the following mathematical model has been used to estimate the two angles [39]:

(1) $$\begin{array}{c} \Theta =tan^{-1}\left(\frac{A_{x}}{\sqrt{A_{y}^{2}+A_{z}^{2}}}\right)\\ \Psi =tan^{-1}\left(\frac{A_{y}}{\sqrt{A_{x}^{2}+A_{z}^{2}}}\right) \end{array}$$

where Θ and Ψ represent the vehicle pitch and roll angle respectively. $A_{x}$, $A_{y}$ and $A_{z}$ represent the static acceleration components of each axis. To evaluate the potential uncertainty associated to the angle values obtained from acceleration data comprising the sensor self-noise, a Monte Carlo (MC) analysis was performed. Acceleration data on each axis were modeled as normal distributions centered on the ideal acceleration values and characterized by a standard deviation of 0.018 g (representing the sensor self-noise provided in the sensor datasheet). A total of $10^{7}$ iterations was performed in the MC test, yielding a pitch angle expanded uncertainty (gaussian distribution, coverage factor 2, confidence level 95.44%) of 0.21 degrees when considering instantaneous acceleration values. By averaging the acceleration values over a window of 20 samples, a reasonable time frame given the transient nature of the application and the temporal dynamics of the phenomenon, i.e., vehicle running, the resulting expanded uncertainty reduces. This averaging process mitigated the acceleration noise, resulting in an angle expanded uncertainty (gaussian distribution, coverage factor 2, confidence level 95.44%) of 0.05 degrees. This results highlight the significant impact of sensor self-noise on the prediction of the instantaneous angle. In addition to the MC analysis an experimental test to measure the angle uncertainty has been conducted by using a certificated sine bar and standardized Johnson Gauge Blocks to generate height differences and hence angular differences (see Figure 6). Four smart sensing nodes have been attached and geometrically aligned on the sine bar and the test has been performed in a metrological room on a vibration-isolated optical table under controlled environmental temperature conditions. The test focused on small angles, i.e., approximately in the range ±3 degrees, as the angles involved in the problem are lower than 1 degree. This test aimed to assess the sensitivity, linearity and repeatability of the angle measurement. The results discussed in Section 3 are obtained by averaging the data provided by the four devices and properly calculating their standard deviations.

### 2.6. Test Case Scenario

**Figure 7:**
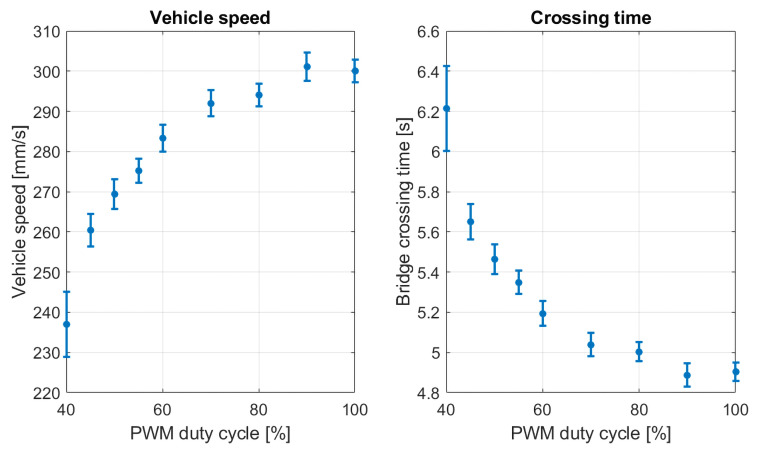
Vehicle speed and crossing time curves for different PWM values.

The experiment involved 90 vehicle crossings over the bridge at varying speeds. Speed adjustments were achieved by modulating the PWM (Pulse Width Modulation) signal duty cycle, ranging from 40% to 100%, subdivided into 9 steps with 10 passages at each speed increment. Throughout the bridge deck, the vehicle speed was maintained constant. As illustrated in Figure 7, a nonlinear correlation between speed and PWM duty cycle was observed. Subsequently, the problem was conceptualized as quasi-static, independent of the vehicle speed, as detailed in the subsequent section. The experiment conclusively established the problem independence from the vhicle velocity.

### 2.7. Mechanical Model

**Figure 8:**
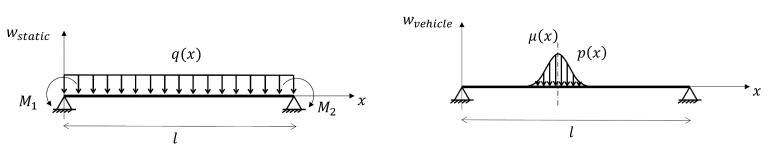
(**left**) Euler-Bernoulli beam theory of a distributed load. (**right**) Specific load positioned at any point along the beam.

In the realm of Vehicle-Bridge Interaction (VBI) studies, a widely adopted model involves a mass moving along the bridge, featuring a spring-damping system that separates the point of contact between the mass and the structure. This model provides a simplified representation, capturing the filtering effect of the vehicle suspensions and the inertia effect of the vehicle mass in relation to the bridge mass [21]. In the context of this study, where the vehicle model does not include suspensions and the problem is treated as quasi-static, the theoretical bridge model is further simplified to resemble a pinned-pinned beam model, with a force propagating along the bridge. The mechanical behavior in this investigation is described as quasi-static, i.e., it is assumed to be unaffected by the vehicle speed and reliant solely on the vehicle position along the bridge. The model exploited is the classical Euler-Bernoulli beam model, which is divided into two components: one accounting for the pre-deformation of the bridge due to its weight and constraints, and the other arising from the passage of the vehicle. Analysis of the collected data revealed that railway track introduces some non-linearities in the bridge model: (1) distributing the vehicle weight on a wider area unloading the bridge structure when the vehicle is close to the bridge constrains, (2) adding an additional static momentum in correspondence of the bridge constrains (see Figure 8).

Consequently, the vehicle weight has been modeled as distributed according to a normal distribution with the mean value μ(x) located in the vehicle center of gravity. A scale factor SF was then used as follow so that the integral of the distributed load correspond exactly to the total vehicle load $P_{vehicle}$. The load distribution on the bridge for each time step has been calculated as p(x) considering the following mathematical formulation:

(2) p(x)=N(μ(x),σ)SF

(3) $$SF=\frac{P_{vehicle}}{\int_{-\infty }^{\infty }p\left(x\right)dx}$$

Afterwards, the Euler-Bernoulli partial differential equation (see Equation (4)) have been numerically solved, for each train position among the 1500 virtual positions considered over the bridge span, exploiting the solve_bvp Scipy Python library function. The following boundaries conditions have been considered for solving the model: w(0,l)=0, $w^{\prime \prime }\left(0,l\right)=0$.

(4) $$EI\frac{d^{4}w_{vehicle}\left(x\right)}{dx^{4}}+N\left(\mu \left(x\right),\sigma \right)\;SF=0$$

where *E* and *I* represents the Young modulus and the section inertia of the bridge. $w_{vehicle}\left(x\right)$ is the bridge displacement due to the vehicle load. The best σ value, representing the vehicle load dispersion due to the rail track interaction, has been obtained by fitting the model using the mid-span displacement sensor data over all 90 passages. Therefore the following cost function has been optimized by using a lest-square optimizer:

(5) $$\sigma_{opt}=\underset{\sigma }{\mathrm{argmin}}\;\;{∥w_{vehicle}\left(l/2,\mu \left(x\right)\right)-w_{exp}\left(\mu \left(x\right)\right)∥}_{L2}$$

where ${∥.∥}_{L2}$ the L2-norm of the difference between the vector containing the experimental values $w_{exp}$ and the vector containing the mid-span displacement from the Euler-Bernoulli model. The μ(x) value therefore changes along the bridge, simulating the passage of the vehicle. The real data were interpolated and fitted to the same points used in the discretization of the bridge length. The static predeformation component of the bridge was modeled as the sum of the deformation caused by the bridge self-weight *q* and two static moments applied to the constraints due to the force exerted by the rail-track on the bridge abutments. The Euler-Bernoulli linear model has been then calculated in the closed form solution as follow:

(6) $$w_{q}\left(x\right)=-\frac{q}{24EI}(x^{4}-2lx^{3}+l^{3}x)$$

(7) $$\begin{array}{c} \begin{array}{c} w_{M1,M2}\left(x\right)=\frac{1}{EI}[\frac{M_{1}+M_{2}}{6l}x^{3}+M_{1}\frac{x^{2}}{2}-\left(\frac{4M_{1}+M_{2}}{6}\right)xl] \end{array} \end{array}$$

(8) $$\begin{array}{cc} w_{static}\left(x,M_{1},M_{2}\right) & =w_{q}\left(x\right)+w_{M1,M2}\left(x\right) \end{array}$$

where $w_{q}\left(x\right)$ is the bridge displacement due to the bridge-self weight load, $w_{M1,M2}\left(x\right)$ is the bridge displacement due to $M_{1}$ and $M_{2}$ which are the unknown torque acting at the constrain level due to the rail-track effect. The model has been compared to the data collected by the vision system, which measures the total vehicle displacement due to the static deformation of the bridge and the bridge deformation due to the vehicle weight. The resulting optimization function results in:

(9) $$\begin{array}{c} M_{1,opt},M_{2,opt}=\underset{M_{1},M_{2}}{\mathrm{argmin}}∥w_{vehicle}{(\mu \left(\left(x\right)\right)+w_{static}\left(x,M_{1},M_{2}\right)-w_{cam}\left(x\right)∥}_{L2} \end{array}$$

where $w_{vehicle}$ is the displacement of the bridge corresponding of the vehicle position and $w_{cam}$ is the displacement measured by the vision system. The simplified Euler-Bernoulli model surely provides a framework for understanding the physical behavior of the system, yet it oversimplifies some of the complexities inherent in the real system. Indeed, if on the one hand this model offers a conceptual understanding of the system, on the other hand it fails to accurately capture the nonlinearities introduced by the interaction of the bridge with the rail-track. Data-driven approaches can help tackling these shortcomings and therefore providing a more accurate model of the vehicle-bridge interaction. This approach aims to overcome the limitations of the Euler-Bernoulli formulation previously discussed by leveraging the flexibility and scalability of complex algorithms. Moreover implementing these approaches directly on the smart sensing node offers a practical solution for scalability and integration into an efficient IoT infrastructure. To achieve this, a training, validation, and test dataset has been built considering the *X* feature matrix containing angle data extracted by the sensor mounted on the moving vehicle passing over the bridge, and a corresponding output *Y* matrix containing displacement data obtained from the vision system for each passage. Due to the limited number of train passages over the bridge, a data augmentation has been applied to the original dataset passing from 90 to 9000 bridge crossing. For each passage a bootstrapping techniques has been used to extract 100 random sub-samples and re-interpolating them in order to have the points corresponding to the same position along the bridge span. This data augmentation process is crucial for regularizing the model and preventing overfitting.

The model proposed for fitting the data is a feedforward neural network considering selected for its simplicity and adaptability for edge detection deployment. The ReLU activation function was applied to each hidden layer. ReLU is widely favored as an activation function in neural networks due to its simplicity, computational efficiency, and effectiveness in addressing challenges like the vanishing gradient problem and inducing sparsity. The linear activation function was used for the final output layer, following standard practice for regression problems. The model input considers the vehicle pitch angle Θ and as output the bridge mid-span displacement $\hat{w}$. The neural network training loss function, denoted as $L_{NN}$, consists of two components. First, it includes the L2-norm error between the predicted displacement $\hat{w}\left(x\right)$ and the displacement measured by the camera $w_{cam}\left(x\right)$. Second, it incorporates a regularization term that promotes physical consistency, represented by the L2-norm error between the neural network prediction and the previously established Euler-Bernoulli model. The regularization term λNN is treated as a hyper-parameter to be optimized during the model tuning process. In this sense, the approach should be considered among the class of Physics Informed Neural Network models.

(10) $$L_{NN}=∥\hat{w}\left(x\right)-w_{cam}{\left(x\right)∥}_{L2}+\lambda_{NN}∥\hat{w}\left(x\right)-w_{static}\left(x\right)-w_{vehicle}{(\mu \left(\left(x\right)\right)∥}_{L2}$$

The dataset, comprising 9000 bridge crossings, was divided as follows: 40% for testing, 36% for training, and 24% for validation. The trainig process considers 5000 epochs for the model training. Early stopping technique has been then used to monitor the validation loss and stop the training thus preventing model overfitting. The early stopping function considers a “patience” value of 15 consecutive epochs to avoid undesired model training stop due to validation loss noise. The max epochs value results be to sufficiently high for the considered dataset considering it has been never reached during all training processes. The neural network model has been trained 1000 times. The standard random weights initialization has been considered for each training session. A Bayesian Optimizer has been used to optimize the model hyperparameters, as detailed in Table 1. The optimizer loss function $L_{BO}$ aimed to minimize the total distance between the predictions $\hat{w_{i}}\left(x\right)$ and the actual data $w_{icam}\left(x\right)$ for all crossings in the test dataset.

(11) $$L_{BO}=\sum_{i=0}^{test}{∥\hat{w_{i}}\left(x\right)-w_{icam}\left(x\right)∥}_{L1}+\lambda_{BO}MACC$$

Furthermore, for a given neural network architecture, it is possible to estimate the Multiply-Accumulate (MACC) value, which is correlated with the MCU flash and RAM memory usage, as well as the model processing time. Consequently, have been added to the loss function a penalization term related to the MACC, aiding in identifying the best model in terms of accuracy and edge processing performance. The amount of penalization have been controlled by the $\lambda_{BO}$ reasonably choosen based on the total error value as 2 × 10^−5^. When two models exhibit similar accuracy, the lighter one is preferred, considering its deployment at the edge. Optimization can also be constrained to match the hardware specifications. In this specific case, tests revealed that for a simple feed-forward neural network, the MACC limit value has been set to 500,000. Beyond this threshold, the model cannot be deployed on the MCU due to exceeding available memory. Quantization, although not considered in this case because for such simple model architecture does not give a real advantage, and it typically helps in reducing memory usage at the cost of a slight decrease in model accuracy.

Ultimately, the optimal model was deployed on the MCU to assess its real-world performance in terms of memory usage and execution time. This evaluation has been carried out using STMicroelectronics tools, which facilitated the simulation of network performance at the edge.

## 3. Discussion and Results

**Figure 9:**
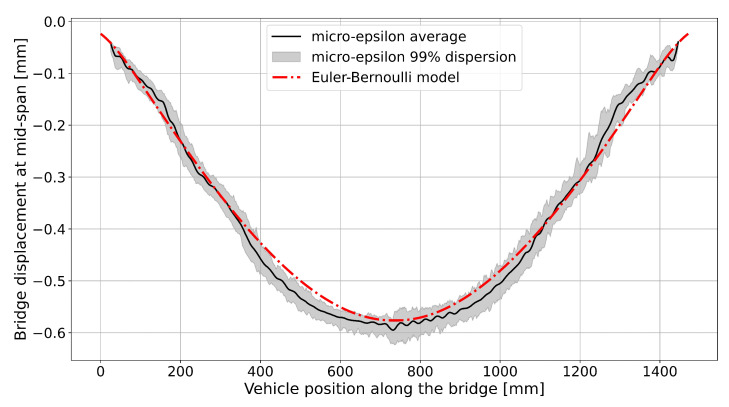
Euler-Bernoulli model fitting on the mid-span displacement.

**Figure 10:**
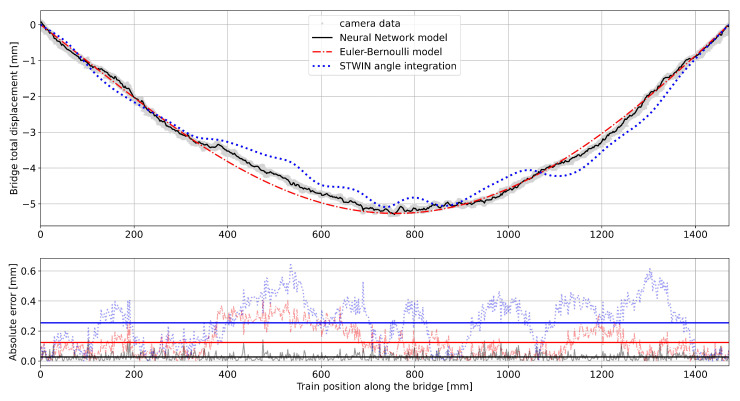
Bridge displacement prediction result. (**Top**) Bridge total displacement for the three models tested. (**Bottom**) Absolute error of the three models with respect to the camera data. Average absolute error as horizontal lines.

The dataset generated by the reference displacement measurements performed with the laser-triangulation device at the bridge mid-span was averaged and compared to the data of the Euler-Bernoulli model for the moving vehicle (see Figure 9). Despite the real data exhibit imperfect symmetry with respect to the mid-span, the analytical model generally describes the actual behavior of the structure quite well. It can be observed that the displacement begins before the vehicle enters the bridge mid-span, indicating a coupled effect between the bridge deck and the rail track. Moreover, the load transmitted to the bridge deck by the the vehicle weight is also spatially distributed as a normal distribution (see Equations (2) and (3)). The standard deviation value obtained from the optimization process described in Equation (5) is 30 mm. The maximum mid-span displacement measured by the triangulation device is −0.6 mm, closely matching the model prediction of −0.61 mm. The overall displacement of the bridge calculated through the analytical model developed, which considers both the static deformation due to the weights of the bridge itself and the component due to the train passing over, was compared to the displacement assessed by the vision-based system. This comparison is reported in Figure 10 and shows that the model globally approximates well the actual displacement of the bridge. The two momentum obtained from the optimization process (see Equation (9)) results to be $M_{1,opt}=-448.7$ Nmm and $M_{2,opt}=-2339.0$ Nmm. The optimization process converges with a total L2-error ${\hat{e}}_{M_{1,opt},M_{2,opt}}$ (see Equation (12)) of 16.54 mm.

(12) $$\begin{array}{c} {\hat{e}}_{M_{1,opt},M_{2,opt}}=∥w_{vehicle}{(\mu \left(x\right))+w_{static}\left(x,M_{1,opt},M_{2,opt}\right)-w_{cam}\left(x\right)∥}_{L2} \end{array}$$

Considering the absolute distance between the data and the resulting model, the average error along the entire span results in 0.12 mm with a maximum error of 0.41 mm (see Table 2). The overall match of the Euler-Bernoulli model with the real data from the camera confirms that the chosen dimensions, including bridge material, deck section properties, bridge constraints and vehicle mass, have been accurately estimated. Furthermore, this initial model investigation reveals that the bridge behavior exhibits some non-linearities that were not accounted for in the simple beam model. These non-linearities arise from the interaction between the bridge and the rail track, suggesting the need for more sophisticated approaches capable of accurately capturing and describing these nonlinear behaviors. Data driven approaches seem the proper solution to tackle this issue.

**Figure 11:**
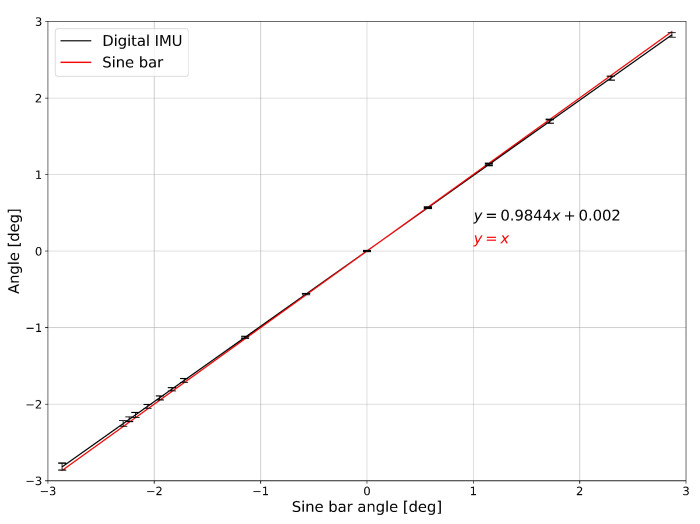
Smart sensing node angle calibration curve and comparison with the reference sine bar.

However, despite these data-driven solutions are very powerful, their prediction accuracy is highly influenced by the quality of the collected dataset. When the dataset is built upon data collected by in-field sensing nodes, their metrological performance have high impact on the final performance of the model. This said, we should recall that the ultimate goal of this study is to develop a real-time bridge deflection prediction model using low-cost sensors, which are susceptible to high noise and non-linear responses. For this reason the target sensors used in this paper underwent a testing and calibration phase, yielding the following results. Angle data from the four smart sensors placed on the calibration sine bar revealed that the smallest detectable angle change is 0.06 degrees. This result is deemed satisfactory, especially if considering the use of accelerometers as tilt sensor for the Θ. This angular value tunrs out to be compatible with the values provided by the MC analysis performed considering data from the sensor datasheet. This also means that the MC can be used to predict the uncertainty of the angle measurement by design, and therefore can represent a valid instrument to properly select the acceleration sensor to be used as clinometer at design level. From the analysis of the calibration curve showed that the sensor behavior in calculating small angles is linear, with minimal deviation from the reference curve provided by the sine bar (see Figure 11). This calibration curve can be used to evaluate the actual angle value by using the linear interpolation curve:

(13) $$angle_{corr}=\frac{angle_{IMU}-0.002}{0.9844}$$

Considering that the expected maximum angle associated to the deformation of the bridge model is less than 1 degree, the angle values obtained from the smart nodes can be used without additional correction, as the maximum error would be around 0.01 degrees. However, for the sake of completeness in this study, all calculated angles were corrected prior to the development of any more intricate algorithms.

**Figure 12:**
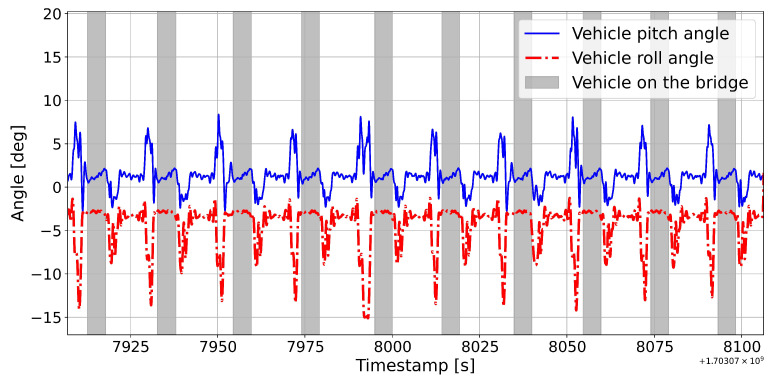
Pitch (blue line) and roll (red dashed line) vehicle angles of 10 consecutive bridge crossing with a PWM value of 45%.

The results related to the smart sensing node installed on the moving vehicle are presented hereafter. Despite applying filtering to the raw acceleration signal at the sensor level, the resulting output angle exhibits considerable noise. This noise is likely attributed to the high sensor noise and the minimal angles characterizing the experimental installation. Nevertheless, as illustrated in Figure 12 and observed across numerous vehicle crossings over the bridge, the angle behavior demonstrates notable repeatability for both the pitch and roll angles of the vehicle. This repeatability presents an opportunity to utilize a significant amount of data and apply averaging strategies, thus diminishing the noise and paving the way for developing a reliable and robust prediction model. Additionally, the plot provides clear insights into the various stages of the circuit: the vehicle ascent to the bridge level while turning and its descent while turning once more (see Figure 12).

To ease the comparison and integration of data from various crossings at different speeds, linear interpolation was exploited to ensure correspondence between acceleration/angle data and spatial locations across the bridge. The bridge span was therefore divided into 1500 equidistant points, maintaining consistency with the previously discussed methodology. Three different models for reconstructing the bridge displacement were tested, each with varying complexity, and their accuracy was compared with the displacement values across the bridge provided by the vision system dataset, which was used as reference in the comparison. The top plot of Figure 10 therefore reports, in addition to the camera data and the data from the Euler-Bernoulli model, the bridge displacements calculated by simply integrating the averaged vehicle pitch angle across the spatial domain using the trapezoid method and by using the feed-forward AI-model previously presented. The pitch angle obtained via data integration exhibits a noisy and oscillatory behavior. This impacts on the displacement evaluated. In fact, while the sensing-node-derived displacement generally aligns well with the reference values, discrepancies can be observed at mid-span. This discrepancy can be attributed to the extremely low angle values characterizing the bridge mid-span when the bridge is deformed. These low values somehow amplify the impact of measurement noise. The Euler-Bernoulli model appears to approximate quite well the bridge displacement. However, some non-linearities in the displacement are not captured by the model. Nevertheless, this model helps understanding how the rail-track can interact with the bridge and which static external forces are applied to the structure. Consequently AI-based model, instead, well reconstructs the bridge displacement based on the angle values collected by the smart sensing node on the train vehicle. This approach performs excellently in predicting the bridge displacement for each passage of the vehicle. This is clearly shown in the bottom plot of Figure 10, where the absolute error between the displacement assessed with the aforementioned approaches is estimated with respect to the displacement measured with the vision-based system. The accuracy results (including average, maximum, and mid-span errors) in estimating the correct bridge deflection, are detailed in Table 2 for the three proposed approaches.

It is worth noticing that the solely use of the angle integration method results in a maximum absolute error that exceeds the bridge deflection caused by vehicle passage (0.6 mm), making accurate detection of the mid-span bridge deflection unfeasible. However, the integration of AI into the smart sensing node significantly reduces the average error, achieving an improvement by an order of magnitude, thereby enabling the detection of the target displacement. The performance of the AI-model were also tested for an edge-deployment. The model uses 70% of the available flash memory (2048 kbytes) and 1% of the MCU available RAM (640 kbytes). The processing time is 27 ms. As the acceleration filtering is performed at the digital MEMS sensor level, it does not consume additional time on the MCU. The angle calculation, exploting the Arm based MCU-optimized CMSIS-DSP functions, requires 2 ms, resulting in a total processing time (filtering + prediction) at the edge of 29 ms. In a real-world scenario, the prediction model could be larger due to the complexity of the bridge-vehicle interaction and the variability within the dataset (e.g, one vs multiple vehicles if considering road bridges). However, hardware configurations can be tweaked to support the deployment of larger and more complex models at the edge (e.g., during the smart sensing node design phase). Moreover, to comply with hardware limitations, various model reduction strategies can be exploited. Techniques such as model distillation and quantization are well-established approaches that enable the deployment of large models on hardware by reducing their computational and memory demands while maintaining high accuracy. Moreover, approaches to split algorithms across different levels, i.e., smart sensing node (smart sensor—aka sensor with processing capability—and MCU), gateway and cloud, could be exploited to scale solutions when addressing multiple vehicles and bridges.

## 4. Conclusions

This paper aimed to discuss the potential of integrating smart sensing into an IoT infrastructure for iBSHM. This integration was demonstrated on a lab-scale set-up reproducing a train vehicle passing over a bridge and by distributing algorithms of different complexities across different hierarchical levels: filtering the signal at the sensor level, calculating the pitch angles at the edge and transmitting the tagged data to a remotely accessible gateway. These data can then be used to populate a database aiming at the development of a bridge health assessment model. Indeed, all the steps leading to the development of this iBSHM solution were tackled in the paper, i.e., from calibrating angle values of low-cost MEMS accelerometer, to developing processing strategies of different complexity, i.e., fitting with analytical (Euler-Bernoulli beam) vs. data-driven models. An important role in the research was played by the developed test-bench, which was redundantly sensorized to ease robust and metrologically valid data collection. The AI-based solution resulted in higher flexibility (better reproduction of non-linearities in the static deflection) and accuracy (maximum error in reconstructing the bridge model static deflection due to the train pass over equal to 0.14 mm) than the analytical model, despite the latter showed quite acceptable results overall (maximum error 0.41 mm). The AI-based approach also resulted light enough to be deployed on the sensing-node MCU, as the overall processing time, including acceleration data filtering and inference, is approximately 29 ms. All these aspects are important to highlight the possibilities opened by the use of these integrated solutions in iBSHM. A final remark is to be performed though. The scaled model targeted in the paper surely does not fully represent a real-world scenario. In fact, in a real world scenario a bridge structure can rarely be simplified as a pinned-pinned beam as well as a vehicle dynamics can affect the accuracy of pitch and roll angle estimations and should be accounted for or mitigated [40]. However, the knowledge and the awareness of these factors help in finding potential solutions to the problem through tests of increasing complexity. The laboratory tests still provide valuable insights for designing innovative iBSHM strategies that leverage advancements in smart sensing nodes, both in terms of processing and communication capabilities. Test set-ups like the one described in this paper make it possible to generate datasets to be used for developing new indirect measurement techniques as well as for comparing performance with more traditional processing strategies. Moreover, as it is clear the advantage of a down-sized solution, it should be also recalled that there are still technical issues in collecting vehicle-to-vehicle and vehicle-to-infrastructure data. This translates in a general and diffused lack of vehicles freely exposing their data to the infrastructure and vice-versa. Contrarily, the availability of vehicle data is essential to leverage the vast amount of available information and monitor whether changes in the collected signals indicate a drift over time, potentially signaling the onset of structural damage. Implementing a predictive model at each vehicle sensor can further enhance this process, as it allows for consideration of individual vehicle behaviors. Treating each vehicle as a distinct entity enables the system to account for these differences, ensuring a more accurate assessment of structural health and improving the reliability of the monitoring framework. The authors are investigating these topics, but these will be the subject of future works.

## Figures and Tables

**Table 1:** Neural Network hyper-parameters tuning.

Parameter	Range	Optimum
n.hidden layers	2–10	9
depth of hidden layers	2–250	15
regularizer $\lambda_{NN}$	0–1	0.002
learning rate	1 × 10^−7^–1 × 10^−2^	0.0041
batch size	8–256	32

**Table 2:** Errors in bridge deflection estimation [mm].

Method	Average	Maximum	Mid Srpan
Angle integration	0.25	0.65	0.21
Euler-Bernoulli	0.12	0.41	0.09
AI-based	0.03	0.14	0.03

## Data Availability

Dataset available on request from the authors.

## Abbreviations

The following abbreviations are used in this manuscript:

SHM	Structural Health Monitoring
iBSHM	Indirect Bridge Structural Health Monitoring
V2I	Vehicle to Infrastructure
IC	Istantaneous Curvature
IoT	Internet of Things
MEMS	Micro Electro Mechanical System
IMU	Intertial Module Unit
MCU	Microcontroller Unit
FPU	Floating Point Unit
ODR	Output Data Rate
FS	Full Scale
LAN	Local Area Network
NIC	Network Interface Card
MC	Monte Carlo
PWM	Pulse Width Modulation
VBI	Vehicle-Bridge Interaction
MACC	Multiply Accumulate
AI	Artificial Intelligence
